# Decellularized extracellular matrices produced from immortal cell lines derived from different parts of the placenta support primary mesenchymal stem cell expansion

**DOI:** 10.1371/journal.pone.0171488

**Published:** 2017-02-02

**Authors:** Gina D. Kusuma, Shaun P. Brennecke, Andrea J. O’Connor, Bill Kalionis, Daniel E. Heath

**Affiliations:** 1 Pregnancy Research Centre, Department of Maternal-Fetal Medicine, Royal Women’s Hospital, Parkville, Victoria, Australia; 2 Department of Chemical and Biomolecular Engineering, Particulate Fluids Processing Centre, The University of Melbourne, Parkville, Victoria, Australia; 3 Department of Obstetrics and Gynaecology, Royal Women’s Hospital, The University of Melbourne, Parkville, Victoria, Australia; University of Sheffield, UNITED KINGDOM

## Abstract

Mesenchymal stem/stromal cells (MSCs) exhibit undesired phenotypic changes during *ex vivo* expansion, limiting production of the large quantities of high quality primary MSCs needed for both basic research and cell therapies. Primary MSCs retain many desired MSC properties including proliferative capacity and differentiation potential when expanded on decellularized extracellular matrix (dECM) prepared from primary MSCs. However, the need to use low passage number primary MSCs (passage 3 or lower) to produce the dECM drastically limits the utility and impact of this technology. Here, we report that primary MSCs expanded on dECM prepared from high passage number (passage 25) human telomerase reverse transcriptase (hTERT) transduced immortal MSC cell lines also exhibit increased proliferation and osteogenic differentiation. Two hTERT-transduced placenta-derived MSC cell lines, CMSC29 and DMSC23 [derived from placental chorionic villi (CMSCs) and *decidua basalis* (DMSCs), respectively], were used to prepare dECM-coated substrates. These dECM substrates showed structural and biochemical differences. Primary DMSCs cultured on dECM-DMSC23 showed a three-fold increase in cell number after 14 days expansion in culture and increased osteogenic differentiation compared with controls. Primary CMSCs cultured on the dECM-DMSC23 exhibited a two-fold increase in cell number and increased osteogenic differentiation. We conclude that immortal MSC cell lines derived from different parts of the placenta produce dECM with varying abilities for supporting increased primary MSC expansion while maintaining important primary MSC properties. Additionally, this is the first demonstration of using high passage number cells to produce dECM that can promote primary MSC expansion, and this advancement greatly increases the feasibility and applicability of dECM-based technologies.

## Introduction

Primary mesenchymal stem cells (MSCs) possess a long list of important properties required for regenerative medicine applications including high proliferative capacity, multi-lineage differentiation potential, ability to deposit extracellular matrix, and capacity to modulate the local immune environment. Primary MSCs of various types are utilized in more than 500 clinical trials including treatments for a variety of disease states such as graft-versus-host disease, bone defects, myocardial infarction, Crohn’s disease and multiple sclerosis [1].

Despite the excitement surrounding primary MSCs, the inability to efficiently produce large numbers of highly functional primary MSCs hinders their utilization. Although primary MSCs can be readily isolated through biopsy from many tissues (e.g. bone marrow, dental pulp, and fat), they are present at very low frequencies [1–5]. For example, 10^5^−10^6^ primary MSCs can be isolated from bone marrow aspirate while the abovementioned therapies require a minimum of 10^8^ highly functional primary MSCs per treatment. To achieve such a large number, the primary MSCs must undergo prolonged *ex vivo* expansion. However, current cell culture technology cannot maintain the phenotype and function of primary MSCs during this expansion process. For instance, after four passages, primary MSCs derived from bone marrow show chromosomal instability, 55% of the cells stop proliferating, and differentiation capacity is significantly diminished [1,6–8]. By the time a sufficient quantity of cells is reached, a large percentage of the MSCs are “filler cells” that no longer possess the desired properties of primary MSCs [6,7,9,10]. This technological shortcoming results in costly and inefficient production of primary MSCs and restricts current clinical use.

Decellularized extracellular matrix (dECM), obtained by removal of primary MSCs cellular components, has recently emerged as a promising but underdeveloped cell culture technology for maintaining primary MSC phenotype during expansion. Recent studies report that culturing primary MSCs on dECM prepared from autologous low passage number primary MSCs, maintains many desirable primary MSC properties during expansion including proliferation and multi-lineage differentiation capacity [9,11–14].

Human bone marrow is the “gold standard” source of primary MSCs for both experimental and clinical studies. Predictably, dECM studies thus far have focused on improving the *ex vivo* expansion of adult bone marrow-derived primary MSC. Bone marrow MSCs (BMMSCs) have the additional advantage of immune privileged/evasive status, which enables allogenic BMMSCs to be used for therapeutic applications without the need for human leukocyte antigens (HLA) matching or subsequent immune suppression therapy [15–20].

However, the use of autologous primary BMMSCs to prepare dECM demands sacrificing a significant portion of primary BMMSCs to provide cells for dECM production. Ng et al. made an important contribution to the field by showing allogenic primary BMMSC-derived dECM also improves the expansion of primary BMMSCs [9]. In that study, dECM prepared from fetal primary BMMSCs acted as a culture surface to support the growth of adult primary BMMSCs. The allogenic dECM matrix was superior to the autogenic dECM as shown by increased primary BMMSC proliferation rates, a smaller cell size distribution, and improved osteo- and adipogenic differentiation capacity. Additionally, these dECM matrices “rejuvenated” several key properties of aged primary BMMSCs. This was an important feature of dECM matrices since the prevalence and function of MSC in the body decrease with age and disease [4,21].

These promising studies highlight the effectiveness of dECM in improving primary MSC expansion. However, several major bioengineering challenges must be overcome before the scientific, commercial and economic potential of this technology can be fulfilled. First, current practice requires that early passage and young primary MSCs produce the dECM, likely due to detrimental compositional changes that occur in the matrix. Using Raman analysis, Sun et al. identified compositional changes that occur within the dECM as a function of MSC age [14]. Additionally, while low passage cells produce dECM that promotes the expansion of primary MSCs, culturing these cells on dECM produced by aged MSCs has been shown to be detrimental to the expansion of the cells [14]. Ng et al. studied the expansion of primary adult BMMSCs on dECM produced by primary fetal MSCs and discovered that after passage 3, the dECM produced by the fetal cells began to lose potency as seen as a decrease in proliferation of the adult MSCs cultured on these surfaces. The necessity to use low passage number and young MSCs to produce the dECM drastically limits the utility of this technology as it requires a continuous source of fresh donor tissue in order to isolate MSCs to produce the dECM substrates [9]. This continual need to isolate, screen and expand primary MSCs from donor tissue is labour intensive, time consuming, and expensive. Additionally, the need to use low passage number cells to produce the dECM drastically limits the total amount of dECM that can be generated. Finally, collection of primary MSCs from tissues of individual donors increases the risk of batch-to-batch variation in matrix quality as well as the risk of disease transfer. Identification of a readily available, reliable and consistent MSC cell type that produces high quality dECM would significantly increase the amount of dECM-coated cell culture surface that could be generated, given the exponential nature of the process.

Placenta and associated tissues are commonly discarded after birth as biological waste, yet they are an abundant, readily available, and ethically acceptable source of allogeneic primary MSCs [16,20,22–27], which are obtained by non-invasive means. Primary placental MSCs have advantages over their BMMSCs counterparts including higher yields of primary MSCs, higher proliferation rates, and possibly a higher “stemness” due to the early embryological origin of the tissue [20,23,24].

At least six different varieties of primary MSCs have been identified in the placenta and associated tissues. Depending on the tissue source, these primary MSCs are of maternal or fetal origin and reside within a vascular or stromal niche *in vivo* [22,25,27–30]. MSCs prepared from the placenta have similar immunophenotype and differentiation potential to other MSCs but they also show distinct properties and behaviours based on their microenvironment of origin (i.e. their niche) [31]. We chose to work with two well-characterized populations of placenta-derived primary MSCs; maternal MSCs isolated from the *decidua basalis* (DMSCs) and fetal MSC derived from the chorionic villi (CMSCs). The basic MSC properties of the primary MSCs are presented in S1 Fig.

We recently reported the establishment and characterisation of immortalized DMSC and CMSC cell lines, referred to as DMSC23 and CMSC29 respectively, which were produced through hTERT transduction. We showed these immortalized cell lines not only maintain their MSC properties (e.g. immnuphenotype and differentiation potential) during *in vitro* expansion (S2 Fig) but they maintained differences in properties and behaviours based on their niche of origin (migration, proliferation ability and resistance to oxidative stress [31,32].

In this study we test the hypotheses that dECM prepared from immortalized DMSC23 and CMSC29 cell lines produced through hTERT transduction will support *ex vivo* expansion of primary MSCs but that the degree of support will differ between the two lines. To test this hypothesis, we cultured primary CMSCs and DMSCs on dECM-coated substrates produced from the two immortal cell lines and measured the proliferation, cell size distribution, and osteogenic differentiation potential of the primary MSCs. Growth on dECM was compared to growth on a monolayer of adsorbed fibronectin, a control dECM produced by 3T3 fibroblasts and conventional tissue culture plastic.

## Materials and methods

### Ethics statement

This study was approved by the Human Research and Ethics Committee of the Royal Women’s Hospital, Victoria, Australia (study number: 14/35). Informed written consent was obtained from all patients prior to delivery. Human term placentae were obtained from healthy women with uncomplicated pregnancies following elective Caesarean section or normal vaginal delivery.

### MSC cell culture

Primary CMSCs were isolated from chorionic villi tissues using the explant method as we described in detail elsewhere [26,33]. Chorionic villi tissues were carefully dissected, minced into small pieces and trypsin-digested (0.25%, Life Technologies, CA, USA) for 40 min at 37°C. Tissues were then allowed to adhere to the plastic tissue culture flasks in Amniomax C100 complete medium (Life Technologies, CA, USA) in a humidified 5% CO_2_ incubator at 37°C.

Primary DMSCs were isolated from *decidua basalis* tissue that remains attached to the placenta after delivery. *Decidua basalis* was dissected from a central cotyledon of the maternal surface of the placenta as described by us in detail elsewhere [33]. Cell suspensions were obtained following overnight 0.25% trypsin and 50 μg/mL DNase 1 (Worthington, NJ, USA) digestion. Following further digestion with 10mg/mL type 1 collagenase (Worthington) and DNase 1, the suspensions were passed through a 100 μm sieve and separated by density gradient centrifugation using Histopaque (Sigma-Aldrich, MO, USA). DMSCs were cultured in α-MEM medium (Sigma-Aldrich) supplemented with 10% fetal bovine serum (FBS, Thermo Scientific, MA, USA), penicillin/streptomycin (100 U/mL and 100 mg/mL, respectively, Life Technologies), and 2mM L-glutamine (Sigma-Aldrich). Adherent cells were passaged after reaching 80% confluence and cells were expanded up to P5.

Prior to any experimentation, both CMSCs and DMSCs were characterized by flow cytometry for primary MSC positive markers (CD73, CD105, CD90, CD146, CD44, and CD66) and negative markers (CD45, CD19, and HLA-DR) as we described previously [33]. Cells were also assessed for *in vitro* differentiation into mesenchymal lineages; adipocytes, osteocytes, and chondrocytes using our previously published methods [33]. Typical immunophenotypic profiles for CMSCs and DMSCs are presented in S1 Fig. 3T3 fibroblasts were cultured in DMEM medium (Life Technologies) supplemented with 10% FBS.

Transduction with hTERT was used to create the CMSC29 and DMSC23 cell lines from the corresponding primary CMSC and DMSC populations as we described previously [32]. The primary MSC characteristics of CMSC29 and DMSC23 cell lines were independently verified for this current study and are presented in S2 Fig [32]. CMSC29 cells were maintained in culture using Amniomax C100 medium and DMSC23 cells were maintained using the Mesencult Proliferation Kit (Stem Cell Technologies, Vancouver, Canada). Primary cells were used up to passage P5 while the CMSC29 and DMSC23 cell lines were used up to passage P25.

### Decellularization of DMSC and CMSC cultures

Decellularized ECM substrates were prepared by a previously reported method [9]. Briefly, CMSC29, DMSC23, or 3T3 cells were plated at a density of 1 x 10^3^ cells/cm^2^ with media change performed every 2–3 days for up to 14 days. From day 3 onwards, 50 μM ascorbic acid (Wako, Japan) was added into the culture medium to increase the production of ECM. To decellularize the deposited ECM, the cultures were washed twice with PBS (Life Technologies) and treated with PBS containing 0.5% Triton X-100 (Thermo Scientific) and 20mM NH_4_OH (Sigma-Aldrich) for 5 min at 37°C. The ECM was washed with PBS, air-dried in the sterile biosafety cabinet and stored at 4°C for up to 1 month. dECM deposited by CMSC29, DMSC23, and 3T3 cells were designated as dECM-CMSC29, dECM-DMSC23, and dECM-3T3 respectively. Fibronectin-coated plates were prepared by incubating tissue culture plates with a 10 μg/mL fibronectin (Life Technologies) diluted in PBS for at least 1 h at 37°C. Excess solution was removed and plates were washed twice with PBS.

### Verification of decellularized extracellular matrices

The decellularization process was monitored by phase contrast microscopy using a Zeiss Axiovert 100 microscope. To verify removal of cell nuclei and DNA, the preparations were stained with DAPI (Vector Laboratories, CA, USA) and visualized using an Olympus IX81 microscope with the appropriate fluorescence filters. Scanning electron microscopy (SEM) was also used to assess the removal of cells. Prior to SEM analysis, specimens were washed twice with PBS and fixed with 2.5% glutaraldehyde (Thermo Scientific) for 30 min. The specimens were dehydrated in ascending concentrations of ethanol (50%, 70%, 90% and 100%). After dehydration, the specimens were sputter-coated with gold-palladium and examined using FEI Quanta 200 SEM.

Dry weight of dECM substrates was prepared from T75 tissue culture flasks of CMSC29, DMSC23, and 3T3 cells. The dECM substrates were detached in deionized water using cell scrapers. The sample weight was recorded after drying in a 70°C oven overnight. The dECM dry weight was normalized to the surface area of the culture plate.

### Immunohistochemical staining of dECM

To explore difference in matrix composition following the decellularization process, immunocytochemistry was performed on dECM-CMSC29 and dECM-DMSC23 coated surfaces. dECM was fixed with 4% paraformaldehyde. Samples were blocked with 5% skim milk powder for 1 h at RT and then washed with 1x PBS to prevent non-specific staining. The surfaces were incubated with mouse anti-human collagen type I (1/400 v/v, Santa Cruz Biotechnology) followed by donkey anti-mouse Alexa Fluor 488 (20 μg/mL; Life Technologies) secondary antibody. To detect the localization of fibronectin, rabbit anti-human fibronectin antibody (0.8 μg/mL; Santa Cruz Biotechnology) was applied, followed by goat anti-rabbit Alexa Fluor 568 (4 μg/mL; Life Technologies) secondary antibody. Staining was visualized using a Nikon A1R microscope and the resulting images were composited by NIS Elements software (Nikon).

To visualize proteoglycans deposition, dECM-CMSC29 and dECM-DMSC23 were fixed in 4% paraformaldehyde for 30 min. Specimens were stained with 1% Alcian Blue solution prepared in 0.1N HCl for 30 min and images were taken with Axioskop 2 microscope (Carl Zeiss) with bright field optics.

### SDS-PAGE

dECM extracts were prepared from CMSC29 and DMSC23. Protein concentrations of each sample were measured using the Pierce protein assay (Thermo Scientific) according to the manufacturer’s protocol with bovine serum albumin as the standard. The protein extracts were resuspended in 7.5 μl XT Sample Buffer supplemented with 1.5 μl XT reducing agent (Bio-Rad), heated to 95°C for 10 mins, and centrifuged at 14,000 g for 2 mins. After centrifugation, each sample was loaded into a 1.0 mm 4–12% gradient Bis-Tris Criterion XT Precast Gel (Bio-Rad) along with Precision Plus protein standard (Bio-Rad). Fibronectin (Life Technologies) and Collagen I (Trevigen, MD, USA) were loaded as high molecular weight protein controls. SDS-PAGE was performed at 150V for 100 mins using XT MOPS buffer (Bio-Rad). The gel was stained with Coomassie Brilliant Blue G-250 overnight and destained in deionized H_2_O for 1 hour. Visualization of the bands was carried out on a densitometer (GE ImageScanner III) and the image was taken using ImageQuant TL software (GE Healthcare).

### Primary MSC proliferation on dECM-coated surfaces

To assess cell proliferation, primary CMSCs and DMSCs were seeded at 100 cells/cm^2^ on the dECM-coated surfaces and cultured for 14 days with a change of culture medium every 3 days. On day 14, cells were harvested using TrypLE Express (Life Technologies), stained with 0.4% trypan blue, and counted using a Countess automated cell counter (Life Technologies). To obtain complete removal of cells from the dECM, we treated the cells with TrypLE Express reagent for up to 15 min in 37°C using gentle agitation on a platform shaker to aid cell dissociation. Further, we always check the tissue culture plates/flasks under the phase contrast microscope to monitor the cell detachment before and after the TrypLE treatment. Following the 15 min TrypLE treatment, we generally observe up to 90% cell viability using the Trypan blue exclusion method on cells cultured within dECM, which are comparable to the cell viability on cells cultured within TCP or fibronectin controls. Population doubling level (PDL) was subsequently calculated with the following formula: X = [log_10_(N_H_)–log_10_(N_I_)] / log_10_(2), where N_I_ = inoculum number, N_H_ = cell harvest number, and X = population doublings [21]. Measuring PDL provides a more accurate estimation at the total number of times that the cell populations have doubled since the initial seeding.

### Immunofluorescence staining of the cell cytoskeleton

Multi-colour fluorescence immunostaining was employed to visualize cells. Cytoskeleton analysis was performed after 72 h of culture. Immunofluorescence staining of the actin cytoskeleton and nucleus of attached cells was performed. Cells were fixed with 4% paraformaldehyde for 20 min and permeabilized with 0.1% Triton X-100 in PBS for 5 min. Non-specific binding sites were blocked by incubating the cells in 1% bovine serum albumin (Sigma-Aldrich) in PBS for 30 min. Cells were stained with TRITC-conjugated Phalloidin (Millipore, 1:500 dilution) for 1 h. Nuclei counterstaining was performed by incubating cells with DAPI (Millipore, 1:2000 dilution) for 5 min. All steps were carried out at room temperature. Olympus IX81 microscope was used for fluorescence imaging and the resulting multi-colour images were composited using Cell R Software (Olympus). The cell area (μm^2^) was quantified using Image J image processing software (National Institute of Health, USA) on a per cell basis. Five images were randomly chosen for each substrate and at least ten representative cells were analysed on each image.

### Primary MSC osteogenic differentiation on dECM-coated surfaces

Primary CMSCs and DMSCs were seeded onto dECM-coated surfaces at a density of 3 x 10^3^ cells/cm^2^ and then Mesencult osteogenic medium (Stem Cell Technologies) was added on the following day. After a 14 day induction period, cells were washed with PBS, fixed with 4% paraformaldehyde, and incubated with the fluorescent OsteoImage staining reagent (Lonza, Basel, Switzerland) as per the manufacturer’s instructions. Fluorescence detection was performed at 492/520 nm excitation/emission wavelengths on a FluoStar Optima plate reader (BMG Labtech, Offenburg, Germany). Cells were stained with 2% Alizarin Red S solution (Sigma-Aldrich, pH 4.2) for 30 mins to detect calcium deposits. Slides were visualized using Axioskop 2 (Carl Zeiss) with bright field optics and images were captured on a Nikon DXM 1200C camera.

### Statistical analysis

All experiments were run in triplicates and repeated at least 3 times (n ≥ 3) unless otherwise stated. All quantitative data are presented as mean ± standard deviation. One-way ANOVA test with Tukey’s multiple comparison tests was performed using GraphPad Prism software (version 5.01 for Windows). A p-value of < 0.05 was considered statistically significant.

## Results

### DMSC23 and CMSC29 cell lines produce dECMs with distinct compositions

**Fig 1:**
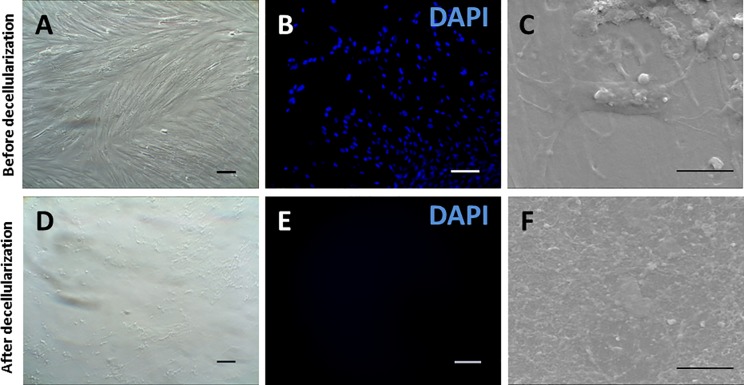
Representative images showing decellularization of DMSC23 cultures. phase contrast (A) before and (D) after decellularization, fluorescence micrographs of DAPI staining (B) before and (E) after decellularization, and scanning electron micrographs (C) before and (F) after decellularization. Scale bar on phase contrast and fluorescence micrographs is 100 μm. Scale bar on SEM images is 5 μm.

CMSC29 and DMSC23 cultures were decellularized according to previously published procedures in order to produce dECM-coated cell culture surfaces [9]. Phase contrast microscopy reveals that the cultures were at confluence before decellularization and that only the ECM scaffold remained after decellularization (Fig 1A and 1D). Fluorescent micrographs of DAPI stained cultures showed well-defined cell nuclei before decellularization and a lack of DAPI staining after decellularization illustrating that the cellular nuclei were disrupted and the genetic material was degraded/removed (Fig 1B and 1E). Scanning electron microscopy also showed the absence of identifiable cells or cellular debris after decellularization (Fig 1C and 1F).

**Fig 2:**
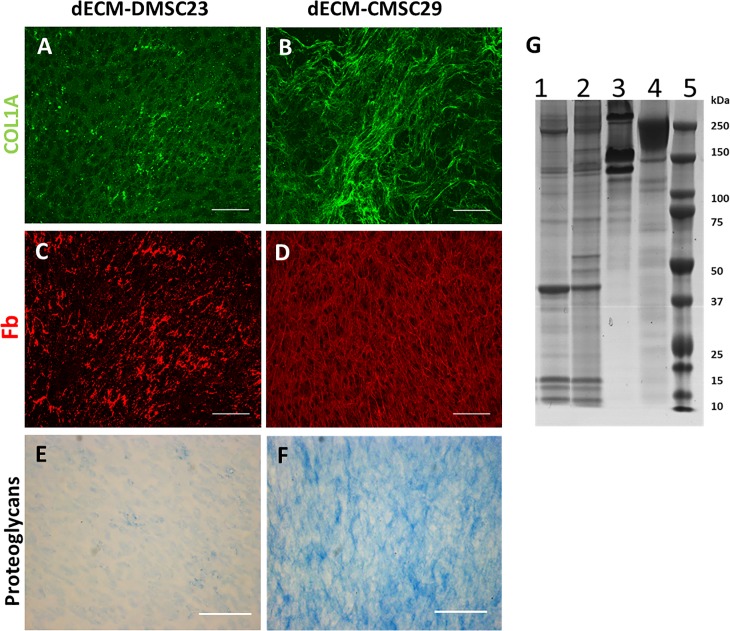
Initial characterization of dECM prepared from DMSC23 and CMSC29 cell lines. Immunofluorescence labelling of collagen type 1 (FITC) in dECM-DMSC23 and dECM-CMSC29 (A and B, respectively). Immunofluorescence labelling of fibronectin (Texas Red) in dECM-DMSC23 and dECM-CMSC29 (C and D, respectively). dECM-DMSC23 and dECM-CMSC29 proteoglycan visualized by Alcian blue (E and F, respectively). Scalebars are 100 μm. (G) SDS-PAGE protein profile of dECM-DMSC23 (lane 1), dECM-CMSC29 (lane 2), collagen I (lane 3), fibronectin (lane 4) and protein standards (lane 5).

To verify that the materials remaining on the cell culture substrates after decellularization were biologically complex ECMs, the surfaces were stained for the common ECM components of collagen type I, fibronectin, and proteoglycans. Fibronectin and collagen type I were selected due to their abundance in ECM. Collagen accounts for more than 90% of the protein in ECM and amongst 25 different isoforms of collagen, the majority of collagen found in ECM is type I collagen. Furthermore, fibronectin is the second most abundant protein in the ECM and it is rich in integrin binding sites that facilitate adhesion to multiple cell types [34]. Both dECM stained positive for each of the ECM components illustrating that the wells were coated with a biologically complex extracellular matrix (Fig 2). Interestingly, the fibronectin and collagen type I produced by CMSC29 and DMSC23 cells showed different relative abundance and distribution throughout the dECMs (Fig 2A–2D). Additionally, Alcian blue staining confirmed the presence of proteoglycans throughout both matrices (Fig 2E and 2f). However, the intensity of Alcian blue staining was higher in dECM-CMSC29 implying that this matrix has higher proteoglycan content.

Matrix characterisation by SDS PAGE analysis was also performed (Fig 2G). Bands correlating in size with fibronectin and collagen type I fragments were present in both dECM-CMSC29 and dECM-DMSC23. However, many other bands were present over a wide range of molecular weights. Distinct banding patterns were observed for dECM-CMSC29 and dECM-DMSC23, which reveals the complex nature of the dECM and biochemical heterogeneity between the matrices.

The amount of dECM produced by the hTERT transduced cell lines was quantified. DMSC23 cell lines produced the most dECM with 738 ± 5 μg ECM/cm^2^, CMSC29 cell lines produced 658 ± 5 μg dECM/cm^2^, while the 3T3 fibroblast control produced the least amount of dECM at 599 ± 39 μg dECM/cm^2^. These data show that all cell lines produce similar amounts of dECM during culture, but the DMSC23 cell line produced the largest amount. Additionally, since all cell types produce similar amounts of dECM, the differences observed in the microscopy images in Fig 2 are likely to be due to differences in composition and spatial arrangement of the biomolecules, not differences in thicknesses of the dECM layers.

### dECM promotes primary MSC proliferation

**Fig 3:**
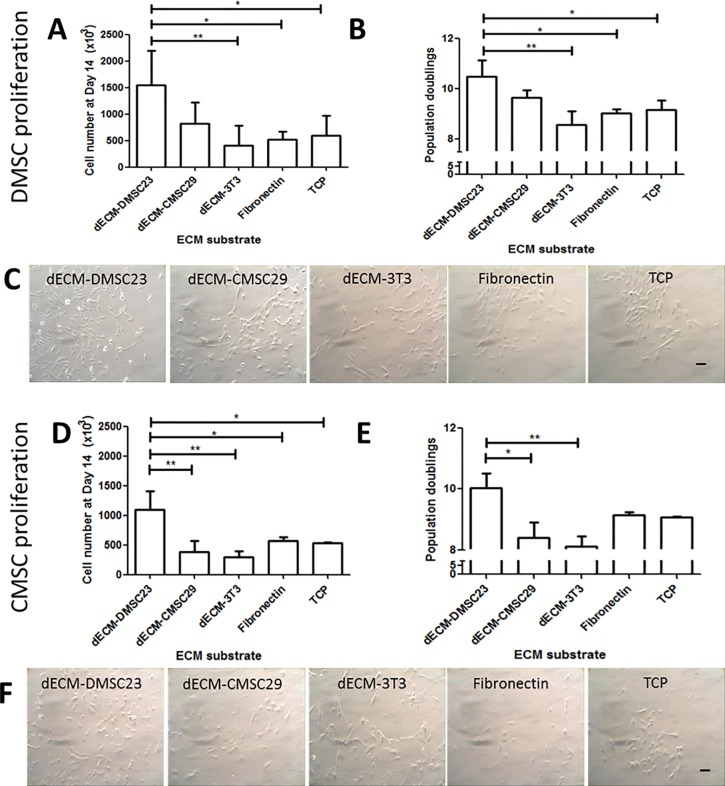
Primary DMSC and CMSC proliferation on dECM substrates. (A) numbers of primary DMSC and (B) numbers of population doublings after 14 days of culture, and (C) representative photomicrographs showing primary DMSC cultured on dECM substrates and control surfaces on day 7. (D) Numbers of primary CMSC and (E) the numbers of population doublings after 14 days of culture, and (F) representative photomicrographs showing primary CMSC cultured on various dECM substrates and control surfaces on day 7. Significant increases in cell proliferation and population doubling levels were observed using one-way ANOVA with Tukey’s multiple comparison test. All values are mean ± SD; n = 3; *p < 0.05, **p < 0.01. Scalebar is 100 μm.

To determine whether DMSC23 and CMSC29-derived dECM promoted the expansion of primary MSCs, cell proliferation was measured after 14 days of incubation. Primary DMSCs cultured on dECM-DMSC23 showed a three-fold increase in cell density at the end of the experiment relative to all the controls (dECM-3T3, fibronectin, and TCP) (Fig 3A). Additionally, CMSCs cultured on dECM-DMSC23 also showed robust expansion, with a two-fold increase over all controls. No increase in primary CMSC or DMSC proliferation was observed when cultured on dECM-CMSC29 (Fig 3A and 3D). These proliferation data were converted into population doublings, which showed that both primary DMSCs and CMSCs had the largest number of population doublings on the dECM-DMSC23 substrate (Fig 3B and 3E).

Phase contrast images of primary DMSCs and CMSCs cultured on various substrates after 7 days revealed an increase in primary MSC proliferation on the dECM-DMSC23 surfaces as shown by greater cell densities (Fig 3C and 3F). We attempted to perform colony forming unit fibroblast assays (CFU-F) [35]. However, we observed that the primary MSCs seeded on the dECM substrates did not form distinct colonies when compared to obvious colony formation on fibronectin-coated tissue cultured plastic or tissue culture plastic alone. Instead, the cells were more evenly spread across the surface of the substrate (Fig 3C and 3F).

**Fig 4:**
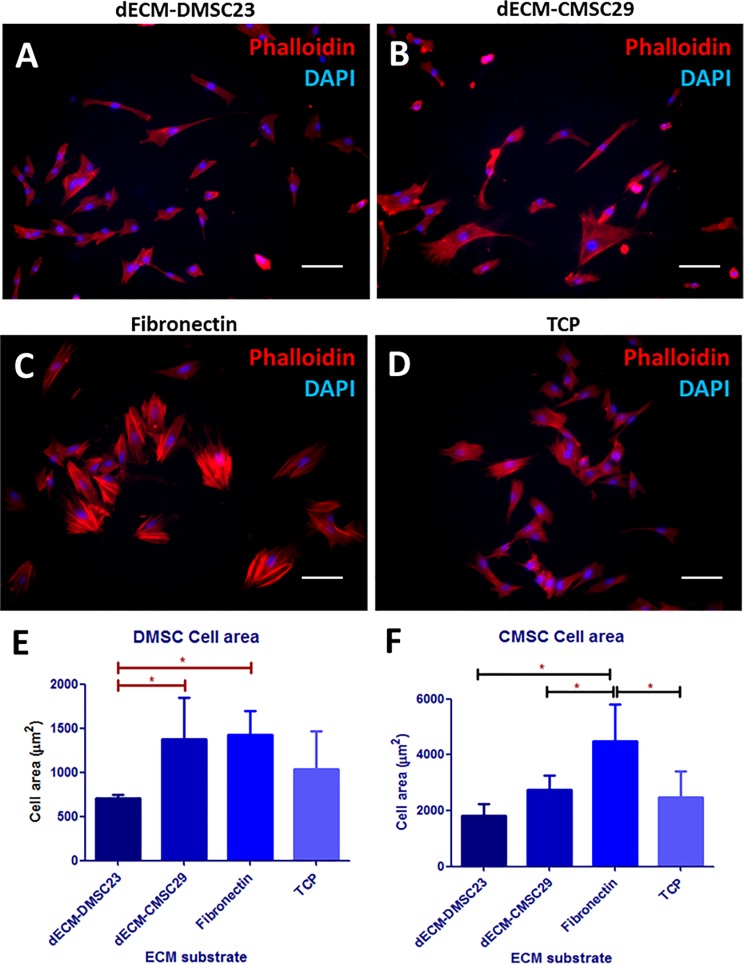
Fluorescence micrographs of DMSC seeded on dECM and control surfaces after 72 h of incubation. (A) dECM-DMSC23, (B) dECM-CMSC29, (C) fibronectin, and (D) tissue culture plastic. Scale bar is 100 μm for all images. (E) Average DMSC cell spread area. (F) Average CMSC cell spread area. All values are mean ± SD, *p < 0.05.

We examined the effect of different dECM surfaces on primary DMSC and CMSC morphology by fluorescence micrography and cell size distribution data in Fig 4. In the micrographs, primary DMSCs are adhered and spread on all of the cell culture surfaces. However, the primary DMSCs and CMSCs on the dECM-DMSC23 substrate were noticeably smaller than those seen on all other surfaces. Others have observed that primary MSCs are smaller at lower passages, and that the cells gradually increase in size during culture. The increase in size correlates strongly with the onset of cellular senescence where the larger cells have exited the cell proliferation cycle [8]. From these data, we infer that the dECM-DMSC23 surfaces can influence DMSC and CMSC spreading, cytoskeletal organization, and overall cell size compared to TCP.

### dECM from hTERT transduced cell lines promotes osteogenic differentiation of primary MSCs

**Fig 5:**
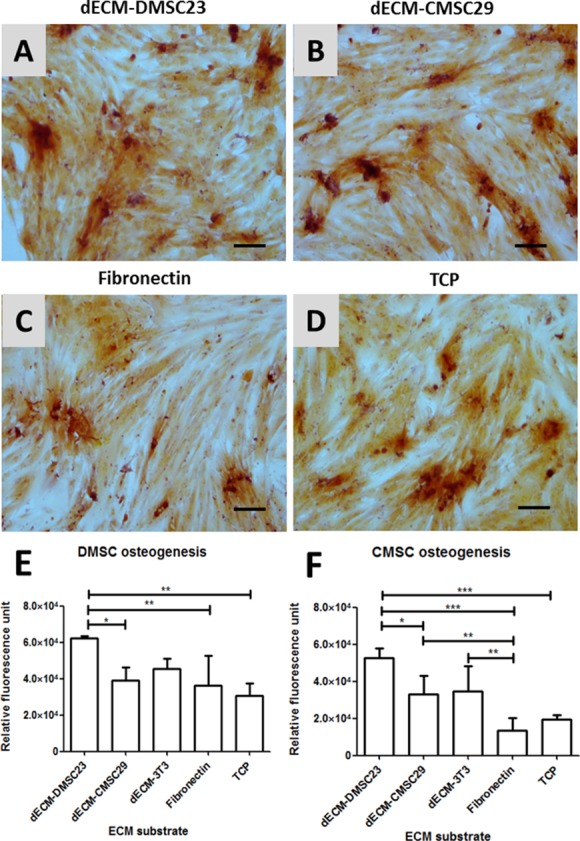
Osteogenic differentiation of DMSC and CMSC on dECM substrates. Representative images of DMSC stained with Alizarin Red S dye after 14 days of osteogenic induction: (A) dECM-DMSC23, (B) dECM-CMSC29, (C) Fibronectin, and (D) TCP. Inset shows control uninduced DMSC. Scalebar is 100 μm. Osteoimage staining in (E) primary DMSC and (F) primary CMSC cultured on dECM and control surfaces after 14 days of osteogenic differentiation. All values are mean ± SD; n = 3; *p < 0.05, **p < 0.01, ***p < 0.001.

We investigated whether there was improved differentiation potential of primary MSCs on the cell line-derived dECM by inducing osteogenic differentiation. After a 14-day induction period, Alizarin Red S staining revealed calcium deposits on all culture surfaces, which shows that the primary MSCs cultured on all surfaces retained their osteogenic differentiation potential (Fig 5A–5D). The degree of osteogenesis was then assessed using the Osteoimage kit, which measures the amount of hydroxyapatite deposition. The results from the osteogenic assay parallel those from the proliferation assay. Primary DMSCs cultured on dECM-DMSC23 exhibited the greatest degree of osteogenic differentiation, yielding a two-fold increase over the TCP control (Fig 5E). CMSC differentiation was also greatest on dECM-DMSC23, with a 2.5 fold increase over the TCP control (Fig 5F). These data illustrate that extracellular matrix derived from the DMSC23 cell line improves the *ex vivo* osteogenic differentiation capacity of two primary MSC types, DMSCs and CMSCs.

## Discussion

Due to the very different tissue niches from which the parent cells for CMSC29 and DMSC23 were derived, the dECMs were expected to have different biophysical/biochemical characteristics and this was borne out in the data presented. DMSCs and CMSCs reside in vascular niches in the maternal *decidua basalis* and fetal chorionic villi, respectively [27,36]. DMSCs and CMSCs in the vascular niche are closely associated with endothelial cells that line the vessel walls. However, the level of exposure to oxidative stress in the two niches is very different. Measurement of the oxidative stress marker, 8-isoprostane, revealed that maternal blood is in a state of higher oxidative state than fetal blood [37]. The proximity of DMSCs to endothelial cells that line the maternal vessels and to the maternal blood circulation suggests that DMSCs are exposed to a high oxidative stress environment. On the other hand, CMSCs are closely associated with endothelial cells that line the fetal vessels and to the fetal blood circulation, which is not subjected to high oxidative stress. Although the chorionic villi are bathed in maternal blood, CMSCs in the vascular niche of the chorionic villi are surrounded by stromal cells and enveloped by two trophoblast layers (i.e. cytotrophoblast and syncytiotrophoblast) and are thus insulates the CMSCs from the high levels of oxidative stress factors present in maternal blood. Biomaterials science has illustrated that various cues are present in the extracellular environment that drive the behaviour of adherent cells. These extracellular stimuli include physical factors such as substrate stiffness and substrate topography, as well as the biochemical composition of the substrate [38–41]. Due to the heterogeneity between the dECM substrates it seems likely that primary MSCs would exhibit differential behaviour when adhered to these substrates. In order to test this hypothesis primary CMSCs and DMSCs were seeded onto dECM-DMSC23 dECM-CMSC29 substrates and their proliferative capacity and osteogenic differentiation potential were assessed.

Proliferation of primary DMSCs and CMSCs on cell line derived dECM was explored. The results presented in this study are consistent with other studies in the dECM literature. The enhanced growth of primary DMSCs and CMSCs on dECM-DMSC23 was consistent with previous literature that show enhanced primary MSCs proliferation on dECM produced by the same primary MSCs type [9,11,12]. For example, Ng, et al. showed that adult primary BMMSCs cultured on dECM derived from fetal primary BMMSCs for 10 days exhibited a 1.6-fold increase in both cell number while in this study we observed a two- to three-fold increase in cell number after 14 days of culture. The most notable difference between these studies is that the dECM used by Ng, et al. was produced by passage 3 primary MSCs (and the dECM began to show loss of activity at passage 4) while hTERT transduced cell lines up to passage 25 produced the dECM used in this work. These results illustrate that hTERT transduced cell lines can be used to produce dECM that still retains the ability to improve the proliferation of primary MSCs. Additionally, the cell lines can produce high quality dECM for up to 25 passages. Due to the exponential nature of cell passaging, and assuming a 1-to-2 split, the ability to culture the cells for an additional 22 passages would theoretically enable (2^22^) millions of times more dECM to be produced from a single source. Furthermore, the beneficial effects of the dECM-DMSC23 were not restricted to primary DMSCs and the dECM-DMSC23 substrate may be generally useful for propagating other primary MSC types.

We investigated the cell morphology characteristics of primary DMSCs and CMSCs grown on hTERT transduced cell lines and found that their cytoskeletal organization and cell spread area were different on each of the surfaces tested. One of the major advantages of using dECM-DMSC23 and naturally-derived matrices for surface coatings is the retention of native ECM architecture and molecular complexity. Therefore, an important factor in the use of cell-derived matrices as biomaterials sources is the selection of the cell types as well as the culture system used to modulate dECM production [34,42].

Our data show that dECM-DMSC23 is similar at promoting the osteogenic differentiation of primary DMSC and CMSC types after osteogenic induction during culture. Our results are consistent with other studies that reported dECM from human and murine BMMSC facilitates expansion, osteogenic differentiation and showed more bone and hematopoietic marrow formation during subsequent transplantation into a murine model [9,12,14,43,44].

The use of transformed cell lines to produce growth surfaces for other cell types is not without precedent. ECM produced by SB623 cells (MSC transduced by NICD vector) supports neural cell attachment and growth [45]. ECM produced by hTERT-transduced human primary MSCs had significant angiogenic potential and promoted neovascularization in endothelial cells [46]. However, to the best of our knowledge, we are the first to utilize hTERT transduced cell lines to provide a long-term source of reproducible matrix for prolonged expansion of primary cells. Additionally, the hTERT-transduced cell lines have additional advantages in eliminating patient-to-patient variation, a challenge normally encountered with primary cell applications. Finally, the hTERT transduced cell lines can be screened for pathogens to minimize the possibility of disease transfer.

The initial cell seeding density needed to produce ECM from DMSC23 and CMSC29 for subsequent dECM preparation was significantly less than that used in other studies that produce dECM from bone marrow primary MSC. Plating 1x10^3^ cells/cm^2^ was sufficient to produce dECM from DMSC23 and CMSC29 after 14 days of culture, whereas other groups reported initial plating densities of bone marrow primary MSC of 6x10^3^-8x10^4^ cells/cm^2^ to produce dECM [12,14,44,47]. Similarly, other studies that employed hTERT-transduced primary MSC to produce dECM reported an initial cell seeding density of 1x10^4^-3x10^4^ cells/cm^2^ [45,46]. This advantage of low initial cell seeding density for subsequent dECM preparation adds to several other advantages of using placenta-derived primary MSCs over their bone marrow-derived counterparts including higher yields of cells, higher proliferation rates, and possibly a higher “stemness” due to the early embryological origin of the tissue [20,23,24]. Our data suggest hTERT transduced placental MSC cell lines such as DMSC23 and CMSC29 may be reliable cell sources for tissue engineering protocols due to their high proliferation rate and ability to produce functional dECM.

In conclusion, we showed that dECM-DMSC23 elicited improved cellular proliferation and osteogenic differentiation of both primary DMSCs and CMSCs. To date, dECM produced by primary MSC from the same source has been used to improve expansion of primary MSCs. However, our data suggest that primary MSCs from different tissue sources (i.e. different niches) may produce superior matrices. The mechanism by which dECM-DMSC23 (but not the dECM-CMSC29) improves the expansion of the primary MSCs needs to be determined. Biomaterials science and basic biological research provide evidence that various cues present in extracellular scaffolding drive the behaviour of adherent cells. These extracellular stimuli include physical factors such as substrate stiffness and substrate topography, as well as the biochemical composition of the substrate [38–41]. In addition to the obvious use of these materials for primary MSC expansion, more extensive analyses of these dECM materials will result in improved knowledge of primary MSC biology through the identification of the novel attributes of the dECM-DMSC23 materials that promote the proliferation and differentiation of primary MSCs. Such studies are beyond the scope of this contribution.

## Conclusion

dECM was produced from two hTERT-transduced, placenta-derived primary MSC cell lines (DMSC23 and CMSC29). Microscopy, immunocytochemistry and SDS-PAGE analyses showed that each dECM had a unique biochemical composition. Primary placenta-derived MSCs cultured on the dECM substrates showed that the dECM-DMSC23 substrate increased primary DMSCs and CMSCs proliferation and osteogenic differentiation. Additionally, the use of the hTERT-transduced cell lines creates the opportunity to produce much larger quantities of high quality dECM without the need to sacrifice the primary MSCs for dECM production. These data support the utilisation of hTERT-transduced MSC lines for the production of larger quantities of dECM, which supports improved expansion of primary MSCs.

## Supporting information

S1 Fig Primary CMSC and DMSC phenotypic characterization. (A-B) Brightfield microscopy images of CMSC and DMSC. (C) Representative photomicrographs showing CMSC and DMSC differentiation into osteogenic, adipogenic, and chondrogenic lineages using Alizarin Red S, Oil Red O and Alcian Blue stainings, respectively. All scale bars are 100 μm. (D) Representative flow cytometry histograms showing DMSC positive expression for CD90, CD146, CD166, CD44, CD73, CD105 and negative expression of CD45, HLA-DR, and CD19. The red histogram shows MSC marker antibody staining while the white histogram shows the corresponding isotype control antibody staining. Additional information on the characterization of the primary CMSC and DMSC can be found in the following citations [31,33].
(TIF)

S2 Fig Transduced cell lines, CMSC29 and DMSC23 phenotypic characterization. (A-B) Brightfield microscopy images of CMSC29 and DMSC23 at P20. (C) Representative photomicrographs showing CMSC29 and DMSC23 differentiation into osteogenic, adipogenic, and chondrogenic lineages using Alizarin Red S, Oil Red O and Alcian Blue stainings, respectively. All scale bars are 100 μm. (D) Representative flow cytometry histograms showing DMSC23 positive expression for CD90, CD146, CD166, CD44, CD73, CD105 and negative expression of CD45, HLA-DR, and CD19. The red histogram shows the MSC marker antibody staining while the white histogram shows the corresponding isotype control antibody staining. Additional information on the characterization of the DMSC23 and CMSC29 cell lines can be found here [32].
(TIF)
