# Supplementary figures and images for: Decellularized extracellular matrices produced from immortal cell lines derived from different parts of the placenta support primary mesenchymal stem cell expansion
Source: PLoS One. 2017 Feb 2;12(2):e0171488. doi: 10.1371/journal.pone.0171488 (PMC5289638; doi:10.1371/journal.pone.0171488)

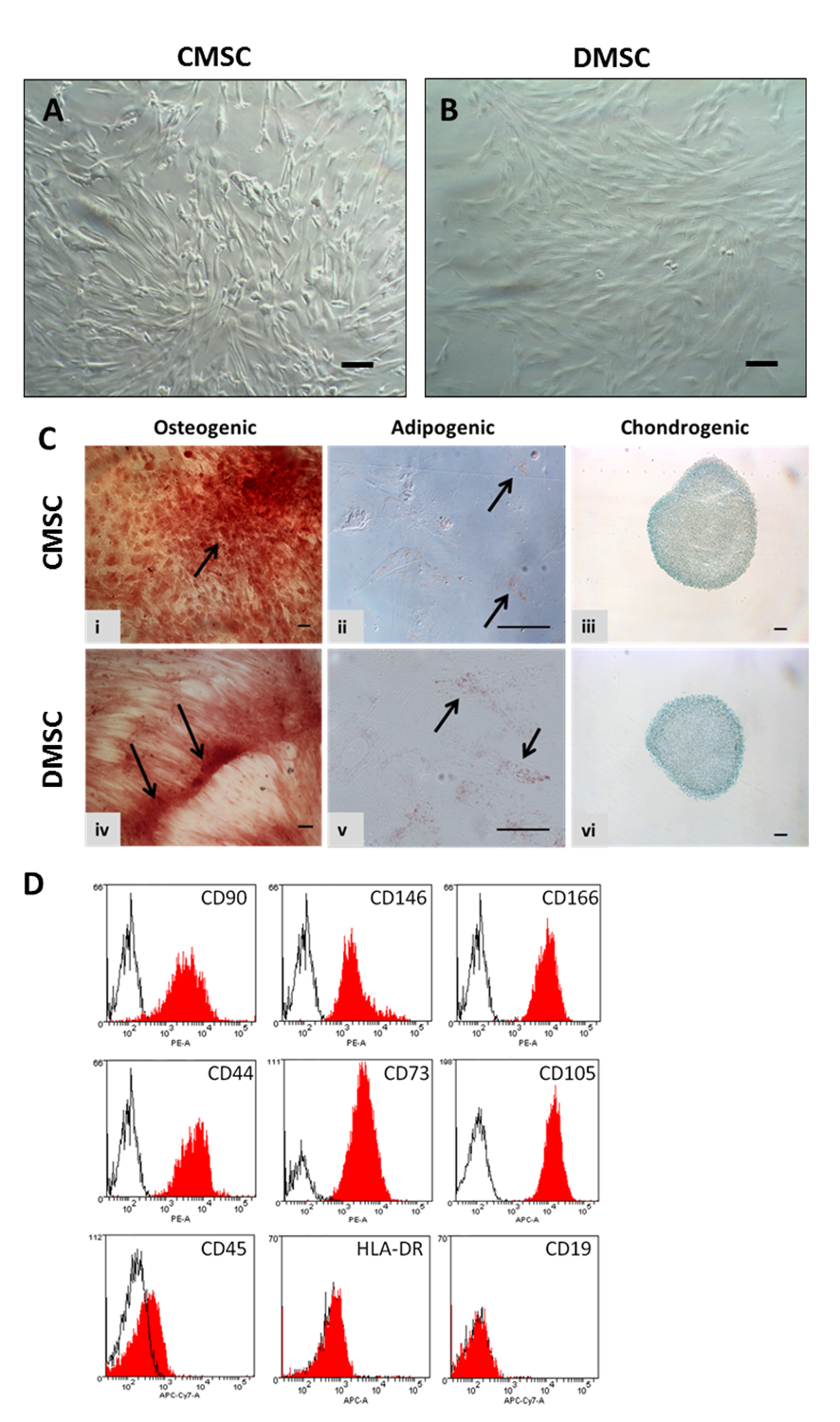

Supplement: S1 Fig — (A-B) Brightfield microscopy images of CMSC and DMSC. (C) Representative photomicrographs showing CMSC and DMSC differentiation into osteogenic, adipogenic, and chondrogenic lineages using Alizarin Red S, Oil Red O and Alcian Blue stainings, respectively. All scale bars are 100 μm. (D) Representative flow cytometry histograms showing DMSC positive expression for CD90, CD146, CD166, CD44, CD73, CD105 and negative expression of CD45, HLA-DR, and CD19. The red histogram shows MSC marker antibody staining while the white histogram shows the corresponding isotype control antibody staining. Additional information on the characterization of the primary CMSC and DMSC can be found in the following citations [31,33]. (TIF) [file pone.0171488.s001.tif]

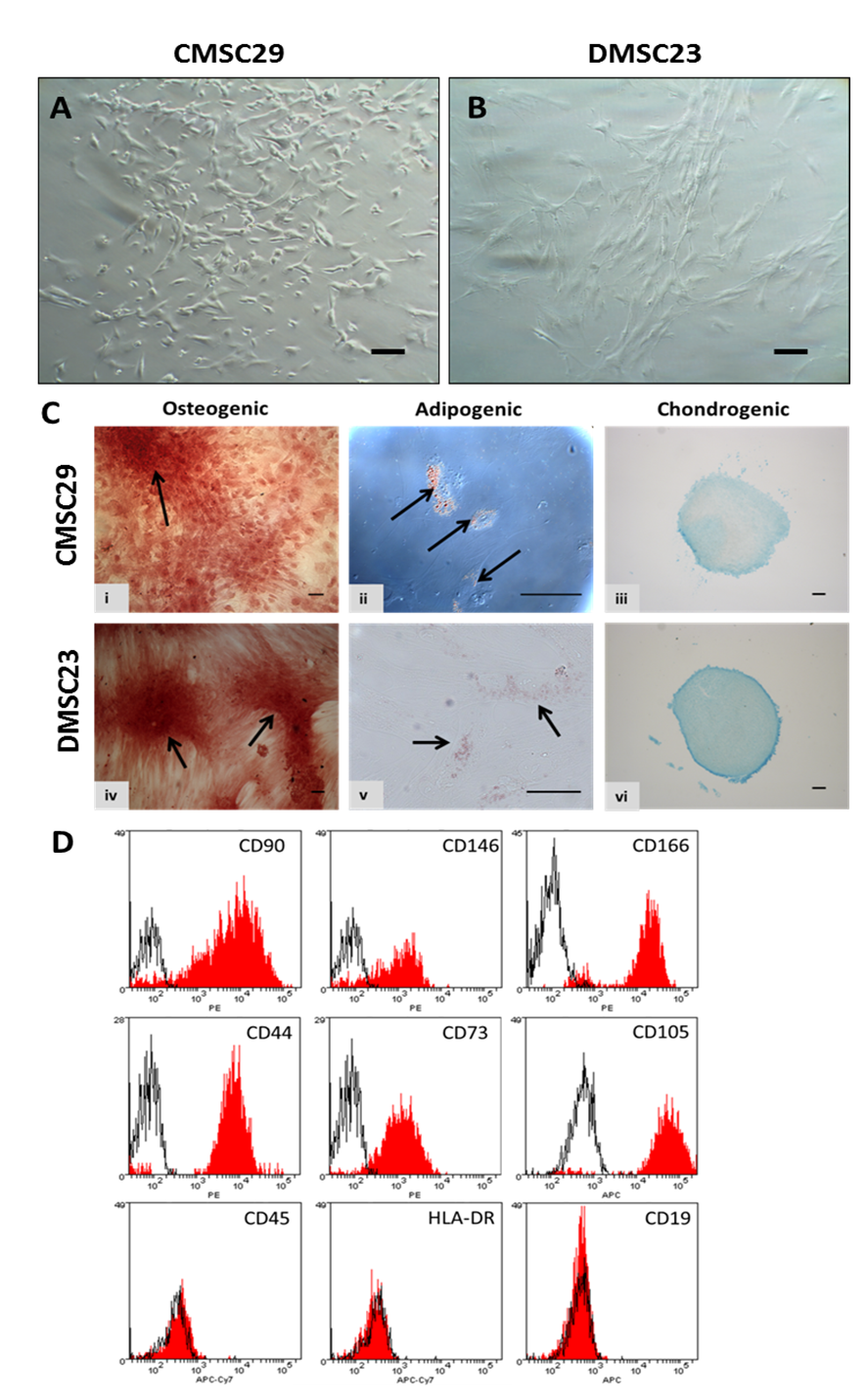

Supplement: S2 Fig — (A-B) Brightfield microscopy images of CMSC29 and DMSC23 at P20. (C) Representative photomicrographs showing CMSC29 and DMSC23 differentiation into osteogenic, adipogenic, and chondrogenic lineages using Alizarin Red S, Oil Red O and Alcian Blue stainings, respectively. All scale bars are 100 μm. (D) Representative flow cytometry histograms showing DMSC23 positive expression for CD90, CD146, CD166, CD44, CD73, CD105 and negative expression of CD45, HLA-DR, and CD19. The red histogram shows the MSC marker antibody staining while the white histogram shows the corresponding isotype control antibody staining. Additional information on the characterization of the DMSC23 and CMSC29 cell lines can be found here [32]. (TIF) [file pone.0171488.s002.tif]
